# Functional Assessment of Patient-Derived Retinal Pigment Epithelial Cells Edited by CRISPR/Cas9

**DOI:** 10.3390/ijms19124127

**Published:** 2018-12-19

**Authors:** Leah P. Foltz, Sara E. Howden, James A. Thomson, Dennis O. Clegg

**Affiliations:** 1 Neuroscience Research Institute, University of California, Santa Barbara, CA 93106, USA; clegg@ucsb.edu; 2 Murdoch Children’s Research Institute, University of Melbourne, Parkville 3052, Australia; sara.howden@mcri.edu.au; 3 Morgridge Institute for Research, University of Wisconsin-Madison, Madison, WI 53715, USA; jthomson@morgridge.org

**Keywords:** retinitis pigmentosa, *PRPF8*, induced pluripotent stem cells, CRISPR/Cas9, retinal pigment epithelial cells

## Abstract

Retinitis pigmentosa is the most common form of inherited blindness and can be caused by a multitude of different genetic mutations that lead to similar phenotypes. Specifically, mutations in ubiquitously expressed splicing factor proteins are known to cause an autosomal dominant form of the disease, but the retina-specific pathology of these mutations is not well understood. Fibroblasts from a patient with splicing factor retinitis pigmentosa caused by a missense mutation in the *PRPF8* splicing factor were used to produce three diseased and three CRISPR/Cas9-corrected induced pluripotent stem cell (iPSC) clones. We differentiated each of these clones into retinal pigment epithelial (RPE) cells via directed differentiation and analyzed the RPE cells in terms of gene and protein expression, apicobasal polarity, and phagocytic ability. We demonstrate that RPE cells can be produced from patient-derived and corrected cells and they exhibit morphology and functionality similar but not identical to wild-type RPE cells in vitro. Functionally, the RPE cells were able to establish apicobasal polarity and phagocytose photoreceptor outer segments at the same capacity as wild-type cells. These data suggest that patient-derived iPSCs, both diseased and corrected, are able to differentiate into RPE cells with a near normal phenotype and without differences in phagocytosis, a result that differs from previous mouse models. These RPE cells can now be studied to establish a disease-in-a-dish system relevant to retinitis pigmentosa.

## 1. Introduction

Retinitis pigmentosa is the most common form of inherited blindness and can be caused by a multitude of different genetic mutations that lead to similar phenotypes [1]. Specifically, mutations in ubiquitously expressed splicing factor proteins are known to cause an autosomal dominant form of the disease, but the retina specific pathology of these mutations is not well understood. In this study, we examined the cellular pathology of splicing factor autosomal dominant retinitis pigmentosa (RP13).

Mutations in ubiquitously expressed proteins provide a unique challenge for understanding pathology. In the case of RP13, it is known that the retina is the specifically affected tissue, but the cellular specificity has not been determined. The two most commonly affected retinal cells are photoreceptors and their supportive retinal pigment epithelial (RPE) cells. To investigate the effect of a patient-specific point mutation in a specific retinal cell type, it is critical to produce a homogenous cell population. In this study, we have produced a purified population of RPE cells to investigate the molecular pathology.

Mutations in three pre-mRNA processing factors are known to cause autosomal dominant retinitis pigmentosa: *PRPF3*, *PRPF8*, and *PRPF31*. Early investigation of 150 Spanish families positively identified specific point mutations in *PRPF3*, *PRPF31*, and *PRPF8* [2]. RP13 is used to refer to the form of the disease caused by one of several known causative mutations in *PRPF8*. The mechanism by which a mutation that affects alternative splicing causes retinitis pigmentosa is unknown [3,4,5].

The human pre-mRNA processing factor 8 (*PRPF8*) gene encodes a protein that is ubiquitously expressed and is one of the largest and most highly conserved nuclear proteins [6]. PRPF8 was first identified as the 220 kDa mammalian homolog of the yeast Prp8 protein, a component of the U5 small nuclear ribonucleotide complex in the spliceosome [7,8]. The role of PRPF8 in pre-messenger (pre-mRNA) splicing has been a topic of investigation for nearly twenty years and has been investigated by a variety of methods to help elucidate the function of this protein in the context of RNA splicing [9,10,11]. Pre-mRNA splicing is critical for the proper removal of introns to allow for subsequent protein translation [12]. Crystallographic studies in yeast have shown that mutations in the *Prp8* gene disrupts protein–protein interactions, but these results have not been confirmed in human protein models [13,14].

RPE cells are highly polarized cells and their function depends heavily on their apical basal polarity. In a functioning retina, the apical microvilli bind and internalize the photoreceptor outer segments. It is possible to assess this function in vitro, which is relevant for modeling RP13. Animal models have shown that the RPE cells of splicing factor knockout mice are unable to phagocytose rod outer segments efficiently [15]. Specifically, RPE cells from *Prpf8* knockout mice were subjected to a rod outer segment phagocytosis assay, and the researchers found a 37–48% decrease in phagocytosis. Using established imaging techniques, it was shown that the cells were deficient in binding of the outer segments rather than internalization [16]. Further examination by immunofluorescence showed that the localization of some adhesion and phagocytosis proteins was perturbed in the *Prpf8* knockout mice. For example, although the αV integrin was correctly expressed on the apical membrane, the β5 integrin and Mertk were expressed throughout the RPE cell in the mutant. Additionally, it was shown that the focal adhesion kinase was localized to the basal side rather than throughout the RPE cells. These findings have led to the hypothesis that RPE cells are the specific cell type affected and the molecular mechanism might involve improper splicing of trafficking proteins [17]. This *Prpf8* mutant mouse phenotype has not yet been shown in humans and studies of disease-specific point mutations have not been investigated.

The patient mutation investigated here is a 6901 C→T missense mutation leading to a proline to serine substitution (P2301S) located in the JAB1/MPN domain in exon 42 of the C-terminal domain of the PRPF8 protein. It has been observed that mutations in the C-terminus of PRPF8 presents an RP phenotype, whereas mutations in the N-terminus are associated with glaucoma [18]. Michael et al. identified the N-terminus variants and suggested that this indicates a clear genotype–phenotype relationship, namely that mutations at the C-terminus may disrupt interactions with BRR2 and at the N-terminus with PRP39 and PRP40 [6,13,19]. A missense mutation at the same nucleotide position (P2301T) was previously reported to cause RP13 [19]. P2301S was first identified in a study of 43 Italian families and was later investigated in the context of the clinical phenotype of one Italian family [20,21]. The pedigree depicts a deceased male that had RP13 with two out of five children suffering from RP13, one of which was deceased and one of which harbored the P2301S mutation. Both of these individuals had children and grandchildren carrying the P2301S mutation, all exhibiting an RP13 phenotype. The disease began with night blindness at an average age of 10.3 years (SD: ±6.4). Fundus examination revealed atrophy of the RPE cells in four living patients, but not in the two younger living patients. Testa et. al. concluded that this mutation results in a mild phenotype with partial preservation of cone photoreceptors, absence of rod photoreceptors, and atrophy of RPE cells [20]. It is difficult to draw any conclusions about the precise cellular pathology from clinical phenotypes, but it is critical to note that both the RPE cells and rod photoreceptors are affected. Cellular modeling of RP13 is necessary to elucidate the cellular and molecular pathology of the disease.

For the purpose of cellular modeling, the Pierce Lab of Harvard Eye and Ear Institute generously gifted RP13 patient fibroblasts to the Thomson lab of University of Madison, Wisconsin. The fibroblasts served as the somatic cell source for producing induced pluripotent stem cells (iPSCs) using an episomal reprogramming strategy as previously described [22]. The patient harbors a P2301S mutation in the gene encoding the splicing factor PRPF8 protein. The patient-specific mutation was corrected using CRISPR/Cas9-mediated gene-editing to generate isogenic-matched control iPSC lines in an efficient new process. The aim of this research is to produce RPE cells from the diseased and gene-corrected patient iPSCs in order to investigate the cellular pathology. We find that RPE cells can be differentiated from both diseased and corrected iPSCs and the resulting RPE cells are similar to wild-type RPE controls in terms of morphology and apicobasal polarity. Furthermore, distinct from previous studies of the mouse knockout, we do not detect differences in phagocytosis between diseased and corrected RPE cells.

## 2. Results

### 2.1. Characterization of RPE Cells

**Figure 1:**
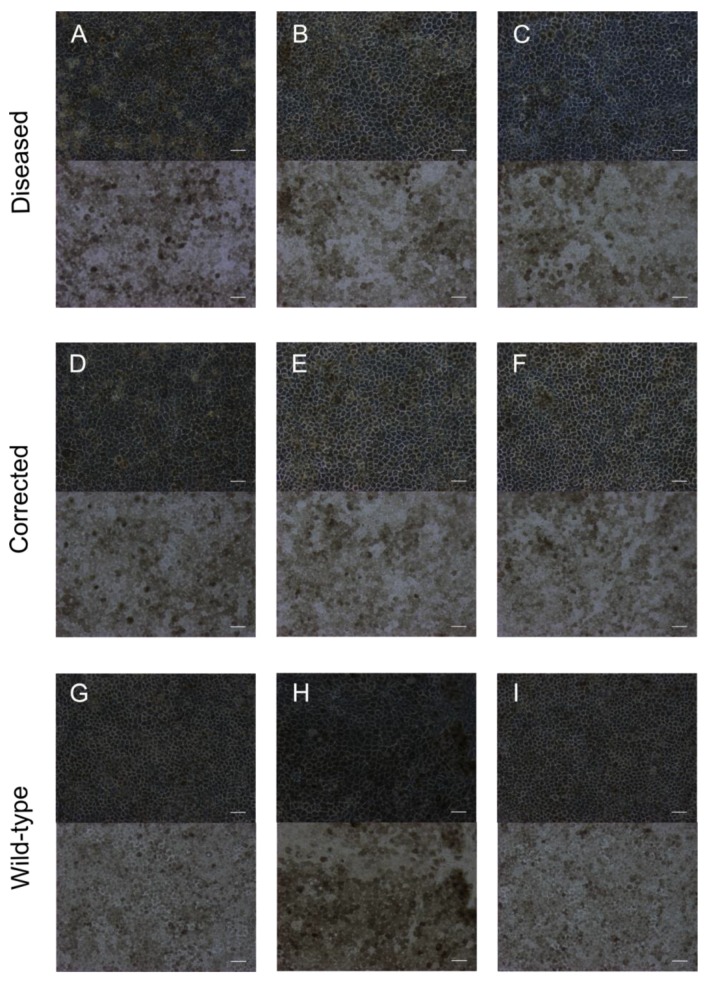
Polygonal morphology and pigmentation at passage 3 on day 30, Fully matured RPE cells exhibit polygonal morphology as observed by phase contrast (20× objective, top images) and pigmentation as observed by bright field microscopy (20× objective, bottom images): (**A**–**C**) iPSC-RPE cells derived from 3 separate patient clones; (**D**–**F**) iPSC-RPE cells derived from three separate patient clones with gene correction; (**G**) H9 embryonic stem-cell-derived RPE; (**H**) MyCell iPSC-RPE cells; (**I**) UCSF4 embryonic stem-cell-derived RPE cells. Scale bars are 100 µm.

**Figure 2:**
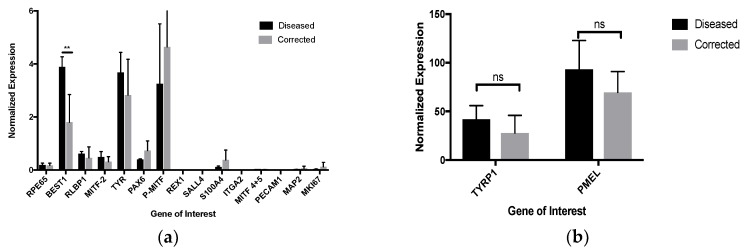

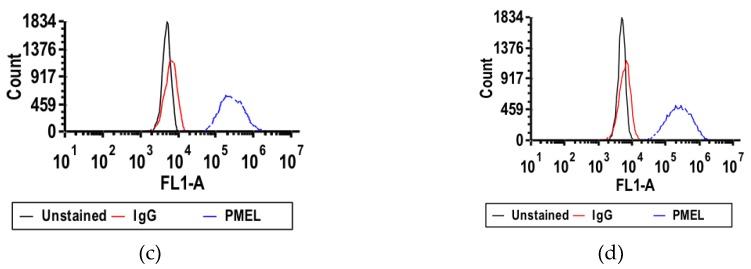
Purified population of PMEL expressing RPE cells and relevant gene expression at passage 0 on day 30: (**a**) measurable expression of RPE-specific genes (*RPE65*, *BEST1*, *RLBP1*, *MITF2*, *TYR*, *PAX6*, and *P-MITF*); (**b**) high expression of *TYRP1* and *PMEL* in immature RPE cells; ns equals not significant, *N* = 3;** *p* < 0.005; (**c**) PMEL expression (blue) in diseased RPE cells as compared to unstained (black) and IgG antibody control (red); (**d**) PMEL expression (blue) in corrected RPE cells as compared to unstained (black) and IgG antibody control (red).

To validate the production of RPE cells from all patient-derived iPSCs, the characterization of the cells was performed by established methods [23,24]. The morphology and pigmentation of the RPE cells was determined by phase and brightfield microscopy (Figure 1). All of the patient-derived RPE cell lines produced polygonal, pigmented monolayers that appear similar to wild-type embryonic stem cells and iPSC-derived RPE cells. To analyze the expression of retinal specific genes, quantitative polymerase chain reaction was performed for several genes. RPE cell genes included *BEST1*, *RPE65*, *PMEL17*, *CRALBP*, and *MITF* isoform 2. Undifferentiated stem cell genes and proliferation markers included *REX1*, *SALL*, and *MKI67*. Non-RPE genes included *S100A4*, *ITGA2*, *MITF* isoform 4+5, *PECAM1*, and *MAP2*. Housekeeping genes included *EIF2B2*, *UBE2R2*, and *SERF2*. All six patient-derived iPSC cell lines were differentiated into RPE cells that expressed all native RPE genes by passage 0 on day 30 or sooner and no longer expressed stem cells or proliferation markers (Figure 2). The diseased cells showed slightly higher expression of *BEST1*, which is implicated in various bestrophinopathies [25]. Interestingly, the only non-RPE gene expressed after differentiation was low levels of *S100A4*, a calcium-binding protein specific to fibroblasts [26]. This may be related to the use of patient fibroblasts as the origin of somatic cells for reprogramming. The purity of the RPE cell population after differentiation was determined by quantifying *PMEL17* expression via flow cytometry (Figure 2).

**Figure 3:**
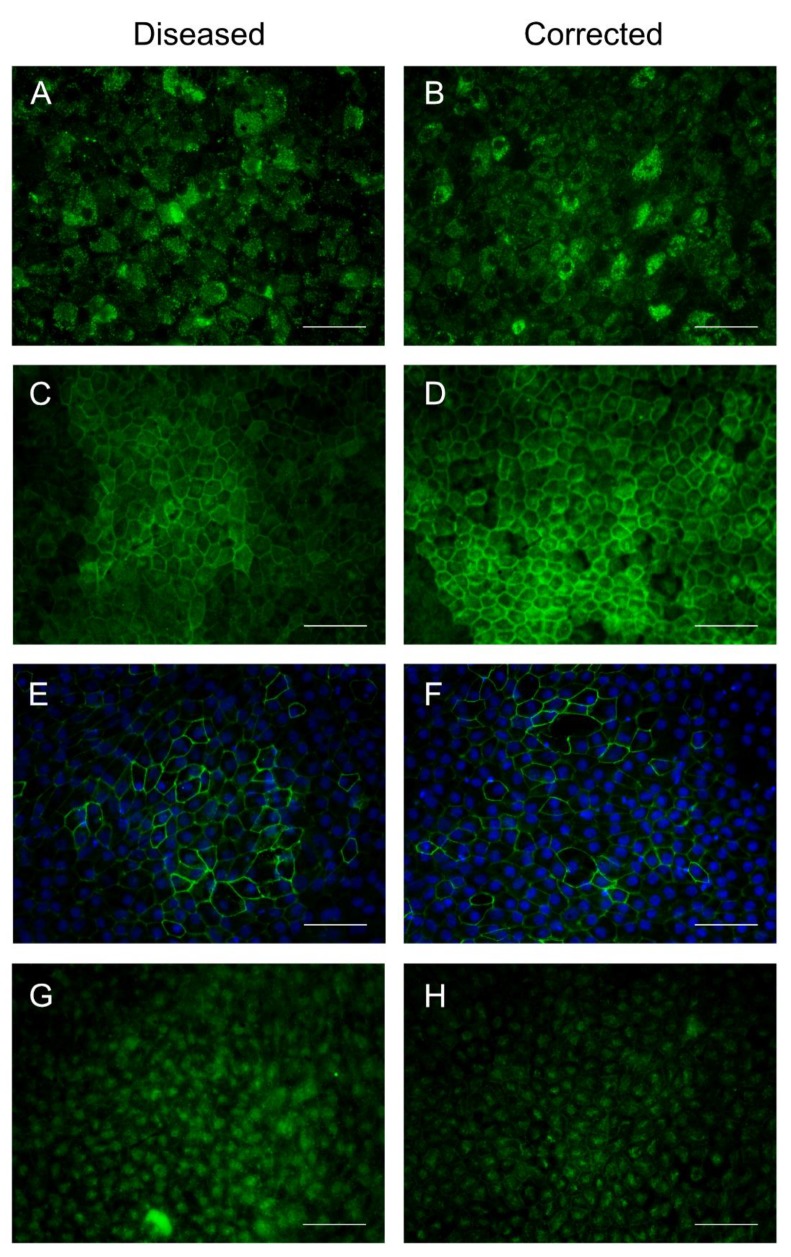
Localization of RPE-specific proteins at passage 3 on day 30. Immunocytochemistry revealed proper localization of RPE-specific proteins and PRPF8 (40× objective): (**A**,**B**) premelanosome 17; (**C**,**D**) bestrophin 1; (**E**,**F**) zonula occludens; (**G**,**H**) PRPF8. Scale bars are 50 µm.

To observe the localization of native RPE proteins, PMEL17, BEST1, and ZO-1 were examined by immunocytochemistry (Figure 3). Premelanosome 17 (PMEL17) is a transmembrane glycoprotein that is expressed early in pigmented cells and serves as an early marker of RPE differentiation [27]. Bestrophin 1 (BEST1) is an integral membrane protein that functions as a calcium-activated chloride channel in which mutations are known to cause retinal degeneration [28]. Zonula occludens (ZO-1) is a tight junction complex that helps establish the integrity of the epithelial monolayer. Diseased and gene-corrected RPE cells expressed these RPE proteins in the correct locations. Localization of PRPF8 was also observed within the nucleus with some potential localization in speckles outside the nucleus.

**Figure 4:**
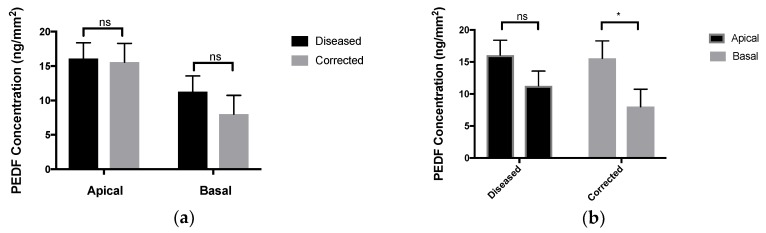
Secretion of PEDF by RPE cells at passage 3 on day 30: (**a**) no significant difference between concentration of PEDF in the apical chamber or basal chamber when comparing diseased and corrected cells; (**b**) significant difference between the apical and basal chambers for corrected cells indicating establishment of apicobasal polarity. ns equals not significant, *N* = 3, * *p* < 0.05.

The secretion of pigment epithelium-derived factor (PEDF) was measured by enzyme-linked immunosorbent assay (ELISA) (Figure 4). The RPE cells were grown on Transwell^®^ inserts to allow for the separation of apical and basal media. For each of the six patient-derived RPE lines, the apical media contained a significantly higher concentration of PEDF than the basal media, indicating the proper polarity of the iPSC-derived RPE cells.

### 2.2. Patient-Derived RPE Cells Are Not Deficient in Phagocytosis

**Figure 5:**
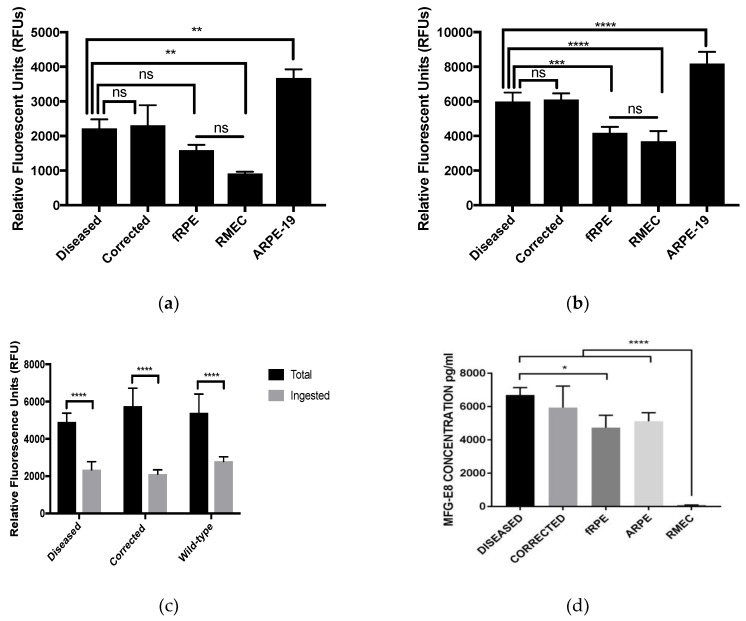
Phagocytosis of photoreceptor outer segments at passage 3 on day 30: (**a**) no significant difference in ingested outer segments between diseased and corrected RPE cells, diseased RPE cells ingested significantly more outer segments than the negative control (RMEC); (**b**) no significant difference in total (bound and ingested) outer segments between diseased and corrected RPE cells, and total outer segments significantly higher than the positive and negative control (fRPE cells and RMECs, respectively). ns equals not significant, *N* = 3, *** *p* < 0.0005, ** *p* < 0.005; (**c**) patient-derived diseased and corrected RPE cells compared to wild-type RPE cells (H9, UCSF4, and MyCell) revealed no significant differences between lines. **** *p* < 0.0005; (**d**) RMECs secreted significantly less RPE-specific MFG-E8. *N* = 3, * *p* < 0.05. RMECs, retinal microvascular endothelial cells; fRPE, fetal RPE; ARPE-19, immortalized RPE cells.

Phagocytosis was measured by challenging the iPSC-derived RPE cells with fluorescently labeled photoreceptor outer segments and quantifying the fluorescence (Figure S3 and Figure 5). The outer segments used in these experiments were purchased commercially to ensure the structural integrity and purity of the outer segments. After five hours of incubation, the relative fluorescent units were measured at an excitation of 488 nm. The total fluorescence comprises both bound and ingested outer segments. A duplicate plate of cells was incubated with Trypan blue without permeabilization to quench fluorescence of any bound outer segments that were not ingested. The results indicate that the diseased and corrected cells were able to bind and ingest outer segments significantly more than the negative control line, retinal microvascular endothelial cells (RMECs). The secretion of milk-fat globule-EGF factor 8 protein (MFG-E8) was measured by ELISA and compared to the same positive and negative control cell lines used in the phagocytosis assay. There was no significant difference between diseased and corrected RPE cells, but both secreted significantly more MFG-E8 than the positive controls (fetal RPE and immortalized ARPE-19) and the negative control, RMECs. Phagocytosis by human fetal RPE (fRPE) cells was lower than the level observed in iPSC-RPE cells. This has been observed in previous studies as well [23,29]. While the difference is not clearly understood, this may reflect the more robust nature of the iPS-RPE cells. Since both diseased and corrected iPSC-RPE cells bound and ingested significantly less than the immortalized ARPE-19, the assay was repeated in comparison to three wild-type stem-cell-derived RPE. All six of the patient-derived RPE lines and the three wild-type (H9 hESC, UCSF4 hESC, and MyCell iPSC) were produced from the same differentiation batch and are shown to phagocytose similar levels of outer segments. All 9 iPSC-RPE cell lines were also assessed for PRPF8 protein expression by the Western Blot and demonstrated similar levels of protein expression (Figure S4).

### 2.3. Atrophy of RPE upon Extended Passage

**Figure 6:**
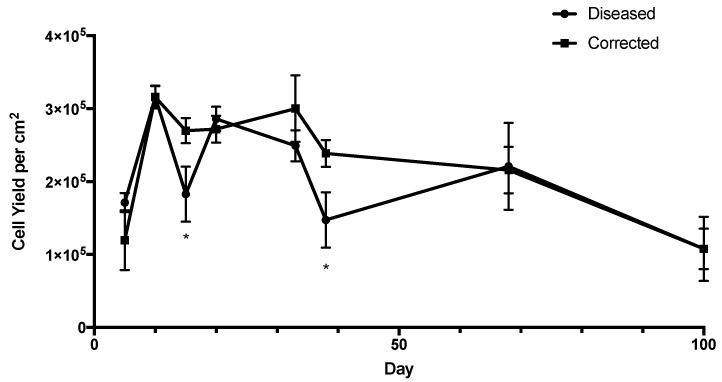
Atrophy of RPE cells upon extended passage. RPE cells were passaged enzymatically at each time point shown at a seeding density of 1 × 10^5^ cells per cm^2^ and allowed growing for 5 to 30 days per passage. Both diseased and corrected RPE cells were able to proliferate until day 100. *N* = 12, * *p* < 0.05.

As a preliminary investigation of atrophy, RPE cells were passaged continuously for 100 days to observe the decline in the number of cells yielded per cm^2^ (Figure 6). After the initial seeding at 1 × 10^5^ cells per cm^2^, the RPE cells were able to reach a density of up to 3 × 10^5^ cells per cm^2^, indicating that the cells underwent approximately 1 to 2 divisions. After six passages, the cell yield started to decline to 2.5 × 10^5^ cells per cm^2^, and by day 100, the RPE cells maintained the initial seeding density, indicating that there was no proliferation occurring. We determine that within 100 days, there was no significant difference in the ability of the diseased and corrected RPE cells to proliferate.

Cells were imaged before passage to observe morphology and pigmentation (Figures S1 and S2). As shown, diseased and corrected cells were able to maintain some level of cuboidal morphology and pigmentation throughout the passages. However, holes and fibroblastic cells started to appear after several passages and pigmentation was sparse. The images shown are meant to be representative, but an unbiased quantification of morphology and pigmentation would be required to draw any conclusions from the cell culture images. As a first estimation, we have relied on cell count to estimate proliferation and atrophy of RPE cells.

## 3. Discussion

With this work, we demonstrate that iPSC-derived RPE cells from a patient, with and without gene correction, can produce a purified population of RPE with morphology, polarity and gene and protein expression similar to native RPE. The patient lines contain the specific disease causing P2301S point mutation in *PRPF8*. The corrected iPSC lines provide a control isogenic RPE cells. Surprisingly, we did not detect differences in phagocytosis between diseased and corrected RPE cells, distinct from previous studies of the mouse knockout model.

After differentiation to a specific cell type, there are several general methods used to confirm the purity and functionality of the cell type of interest: (1) genetic analysis by polymerase chain reaction or next-generation sequencing, etc.; (2) protein analysis by the Western Blot, ELISA, fluorescence-assisted cell sorting, etc.; and (3) functional analysis such as photoreceptor outer segment phagocytosis. The same methods that have been used to confirm differentiation of a particular cell type may also be used to look for abnormalities in a disease state. While it would have been useful to find a difference between the diseased and corrected RPE cells, it is unsurprising that these assays did not reveal any distinction. There is no evidence that a mutation in *PRPF8* affects the expression of RPE-specific genes or protein localization and secretion in human RPE. There was a mouse model that argued that the protein localization of integrin subunits was affected in a knockout mouse for PRPF8, PRPF31, and PRPF3 [15]. There is an important distinction to make between the mouse studies and the human disease. We chose to use patient iPSCs that harbor a missense mutation that is a known causative mutation in human, and therefore more closely models the disease. Our goal was to model human disease, rather than corroborate the results obtained in mouse. We feel that results from a knockout in human cells would not provide valuable information in the context of disease modeling.

The reproducibility of differentiation protocols is vitally important to the success of disease modeling. In order to draw conclusions from any observed differences between cells, there must be a strong indication that the cell populations are similar to those obtained by other measurements, especially considering the concerns about line-to-line variability [30,31]. Once the gene expression and protein localization and secretion have been established, functional assays that have been shown to play a role in disease can be observed. RPE phagocytose photoreceptor outer segments (POS) on a daily basis, which is critical to the health of the retina [32]. The phagocytic relationship between RPE and photoreceptor outer segments was first observed by autoradiography over forty years ago [33]. Modern methods utilize the ability of RPE cells to phagocytose as a measurement of RPE cells health and differentiation efficiency in vitro [34]. RPE cells phagocytose via specific integrin subunits and are significantly more active in phagocytosis than most other cell types, allowing researchers to definitively identify RPE cells in culture [16,35]. Quantification of phagocytosis has been examined by several methods, including pixel detection and fluorescence plate reader [35]. The RPE cells used for functional analyses were matured for up to three passages, with the exception of the extended passaging. As described in Foltz et al. (2017), the standard maturation of RPE for three passages totals twelve weeks at confluence and four weeks at each passage [36]. Stated precisely, passage 0 RPE cells are grown for four weeks, passage 1 are grown for four weeks, passage 2 are grown for only 3–5 days for cryopreservation, and then passage 3 are grown for four weeks [36]. We carried out immunocytochemistry and performed Real-time PCR on a number of RPE markers of maturity, comparing diseased to control iPSC-RPE cells. Functional analysis of RPE cells grown continuously for longer periods, and under a variety of culture conditions, including oxidative stress for example, are worthy of further experiments.

Farkas et al. argued that phagocytosis was deficient in the mutant RPE cells after isolation from the mice, which remained to be shown in human cells [15]. The mutation that was introduced in the mouse model (H2309P) was in the same domain as the patient cells (P2301S). In fact, this subregion of the JAB1/MPN domain of PRPF8 is highly conserved across species and contains seven amino acids that have been recorded to have disease-causing point mutations [2,13,19]. The highly conserved nature of these residues suggests their critical role in the ill-defined function of PRPF8. In order to assess phagocytosis, optimizations were required to observe a significant difference between the stem-cell-derived RPE cells and the negative control cell line. Previous attempts of our phagocytosis assay were performed with preparations of photoreceptor outer segments that were isolated from whole bovine eyes in full light. The resulting preparations resulted in bleached outer segments, whereas commercially available outer segments retained the color of the tissue as it was isolated under low light conditions. Furthermore, the fluorescent labeling was performed at centrifugation speeds that have been reported to shear outer segments. By reducing the centrifugation speeds, outer segments were shown to retain their rod-like morphology. We believe this reduction in bleaching and shearing allowed there to be a significant difference between the negative cell line (RMEC) and the stem-cell-derived RPE cells. We used outer segments for the phagocytosis assays, rather than latex beads, to ensure that the phagocytosis observed in vitro was RPE-specific, as is the standard in the field. We included the RMEC line to control for non-specific uptake, and we did see low levels of non-specific ingestion/binding by these cells. However, the level of phagocytosis by the iPSC-RPE cells is significantly higher, shown both quantitatively and qualitatively (Figure 5 and Figure S3). The most important comparison here is between the diseased and the control iPSC-RPE cells. We see no significant difference. Even if there was some level of non-specific uptake, we would expect to see some difference in diseased versus control if the pathway was impaired. The incubation time is based on established methods of in vitro phagocytosis for RPE cells in culture and their modern modifications for stem-cell-derived RPE cells and disease modeling [16,23,35,37,38,39,40].

The secretion of PEDF is frequently used as a measurement of apicobasal polarity and maturity of the RPE. We also wanted to investigate the secretion of MFG-E8, which is more closely related to the phagocytic function of RPE and had not been previously measured in stem-cell-derived RPE cells by the differentiation method used here [36]. There was no significant difference in the ability of diseased and corrected RPE cells to secrete PEDF, and when compared to negative controls, stem-cell-derived RPE cells secreted significantly more MFG-E8, as expected. We believe that secretion of MFG-E8 and other proteins directly related to phagocytic function may help elucidate pathology of phagocytic defects in other disease models, specifically with regards to the essential roles of MFG-E8, MERTK and integrin αVβ5 [41].

In addition to requiring robust differentiation procedures to produce a homogeneous population of RPE cells and optimization of functional assays, the initial reprogramming of the patient fibroblasts into iPSCs must also be robust. Due to the relative simplicity and high efficiency, CRISPR/Cas9 has been used increasingly for genetic modifications in stem cells. Howden et al. have developed an efficient protocol for the generation of gene-corrected cells lines that undergo the more precise homology directed repair pathway (HDR) rather than the predominate yet more mutative non-homologous end joining (NHEJ) [42,43]. The CRISPR/Cas9 system developed by Howden et al. and used to edit the cells in this study relies on two key factors related to the cell cycle: (1) cells prefer NHEJ in G1 phase and HDR in S phase, and (2) fusion of Cas9 endonuclease and geminin protein (Cas9-Gem) can be used to degrade Cas9 in the beginning of G1 phase. This enables genetic correction of patient mutations with a reduced frequency of insertion/deletion mutations in the untargeted allele [43].

In the context of disease models of inherited retinal dystrophies, several questions remain: (1) whether a given disease primarily affects the RPE or photoreceptor cells, (2) which mouse and/or human models are most appropriate, (3) how to account for ageing and the progressive nature of degenerative disease, and (4) most importantly, how a mutation in a ubiquitously expressed splicing factor causes a retinal disease. The work presented here only investigates the RPE cells, and thus cannot answer the question of pathology in photoreceptors. However, other disease models have revealed similar differences in both cell types, implying that there may not be a clear distinction in pathology. Leber congenital amaurosis as caused by mutations in centrosomal protein 290 kDa (CEP290) has been shown to cause a ciliopathy defect in both RPE cells and photoreceptors [44,45]. With regard to the second question, thus far, we have found that a phenotype observed in a mouse model of RP13, namely phagocytosis deficiency, was not observed in a human model. This difference may be due to the difference in specific mutations, or may be due to differences between non-primate animal retinas and human retinas, especially due to the lack of a macula and variance in cone-to-rod ratios [46,47,48]. Given that the rod cells may be the first cell type to degenerate in retinitis pigmentosa as indicated by the early onset of night blindness, non-primate animal models may be limited to the early phases of degeneration at best [20].

The ability to model degenerative diseases with cultured cells has been called into question since iPSCs may not reflect symptoms that occur in patients until the second, third, or fourth decade of life. However, current successes in disease modeling suggest cellular phenotypes can be detected for a range of degenerative diseases in a matter of weeks to months [25,49]. Furthermore, it may be possible to “accelerate” or mimic aging stress in the dish. Finally, the question remains as to how a point mutation in a ubiquitously expressed splicing factor, such as PRPF8, can cause retinitis pigmentosa. We show here that robust RPE can be reproducibly differentiated from both diseased and corrected iPSCs. We can now examine mRNA expression and in particular mRNA splicing in these cells, which we hope will shed light on this question.

## 4. Materials and Methods

### 4.1. Pluripotent Cell Line Derivation and Maintenance

Human iPSC lines were derived from fibroblasts isolated from the skin biopsy of a patient with a heterozygous c.6901C>T point mutation in the *PRPF8* gene (gift from E. Pierce to J. Thomson, Ocular Genomics Institute, Boston, MA, USA). The fibroblasts were reprogrammed using episomal vectors encoding OCT4, SOX2, NANOG, c-MYC, KLF4, LIN28, and the SV40 Large T-Antigen in addition to a fourth episomal vector containing the miR302/307 cluster [22,50]. For gene correction, a plasmid carrying a PRPF8-specific sgRNA that overlaps the patient-specific mutation, mRNA-encoding Cas9-Gem and an ssODN repair template were introduced into patient-specific iPSCs via electroporation (1100 V, 30 ms, 1 pulse) using the Neon transfection system (Thermo Fisher, Waltham, MA, USA) (Figure S5) [42]. Howden et al. (2014) describes the ssODN sequence, efficiency, and total number of clones screened [50]. Cells were plated a Matrigel-coated 6-well dish in E8 medium with 5 μM Y-27632. The medium was switched to E8 without Y-27632 the next day and changed every other day. Individual colonies were isolated, and expanded, and gene-corrected clones were identified by Sanger sequencing of PCR amplicons generated using primers flanking the patient-specific mutation.

The H9 hESC (WiCell Research Institute, Madison, WI, USA, http://www.wicell.org) and MyCell iPSC line (no. 1013.201, Cellular Dynamics International MyCell iPSC Services, Madison, WI, USA, http://www.cellulardynamics.com) were adapted from mTESR™1 (StemCell Technologies, Vancouver, BC, Canada) and manual passage to TeSR™-E8™ and Versene passage with manual dissection of differentiated colonies. All pluripotent cell lines were maintained on hESC-qualified BD Matrigel (BD Biosciences, Sparks, MD, USA, http://www.bdsciences.com) with daily medium changes and Versene passaging every 4–7 days. All cell lines were cryopreserved in 10% DMSO in TeSR™-E8™ at approximately 2 × 10^6^ cells per mL.

### 4.2. Maintenance of Immortalized and Primary Cell Lines

Primary human retinal microvascular endothelial cells (RMEC; Cell Systems, Kirkland, WA, USA, https://cell-systems.com/) were obtained at passage 4 and expanded to an intermediate cell bank at passages 7 and 9. RMEC were maintained on Matrigel (BD Biosciences) in microvascular endothelial cell media and passaged with trypsin (0.05%; Millipore Sigma, St. Louis, MO, USA, http://www.sigmaaldrich.com) between days 3 and 5 before cells reached confluence. Immortalized human RPE cells, ARPE-19, were maintained in ARPE-19 media: DMEM/F12 with sodium pyruvate and 1× GlutaMAX (Gibco, Waltham, MA, USA), 10% fetal bovine serum (Atlanta Biologicals, Flowery Branch, GA, USA, http://www.atlanta-bioloigicals.com/), and 15 mM HEPES butter. Human fetal RPE (fRPE) (gift from P. Coffey) were maintained on Matrigel in RPE media [51]: MEM-alpha modification (Millipore Sigma, Burlington, MA, USA) was supplemented with fetal bovine serum (5%, 15% for the first 3 days after seeding; HyClone), N1 (1×; Millipore Sigma), NEAA (1×), GlutaMAX-I (2 mM; Invitrogen, Carlsbad, CA, USA), taurine (250 µg/mL; Millipore Sigma), triiodothyronine (0.013 µg/L; Millipore Sigma), and hydrocortisone (20 ng/mL; Millipore Sigma).

### 4.3. Differentiation of Pluripotent Cells to RPE Cells

PRPF8 iPSC lines and wild-type iPSC MyCell and H9 hESCs were seeded onto Matrigel-coated 6-well plates (Corning, Corning, NY, USA, https://www.corning.com/) and cultured for 3 to 7 days before passage by Versene. Directed differentiation was initiated by Versene passage of undifferentiated stem cells to 12-well plates (Corning). Undifferentiated stem cells were left in small clumps rather than in single cells, and thus an exact seeding density of cells per cm^2^ was not possible. Instead, optimization of passage was completed by a serial dilution of stem cells into a 12-well plate and examined for neural projections at day 4 of differentiation.

Growth factors and small molecules were added over the course of 14 days as previously described [24]: DMEM/F12 with 1× B27, 1× N2, 1× NEAA (Invitrogen, http://www.invitrogen.com), 50 ng/mL Noggin, 10 ng/mL Dkk1, 10 ng/mL IGF1, 5 ng/mL bFGF (R&D Systems, Minneapolis, MN, USA, http://www.rndsystems.com), 10 mM nicotinamide (Millipore Sigma), 100 ng/mL Activin A (Peprotech, Rocky Hill, NJ, USA, www.peprotech.com), 10 μM SU5402 (Santa Cruz Biotechnology, Dallas, TX, USA, www.scbt.com), and 10 μM CHIR99021 (Stemgent, Cambridge, MA, USA, www.stemgent.com). If necessary, cells with non-RPE morphology were manually dissected and removed at day 14, all remaining cells with RPE morphology were passaged using TrypLE (Invitrogen) for 5 min at 37 °C and 5% CO_2_ and passed through a strainer with 30 μm wide pores. Immature RPE cells were seeded on Matrigel-coated plates at density (1 × 10^5^ cells per cm^2^) and allowed to mature for 4 to 5 weeks. RPE cells were matured and cryopreserved to create an intermediate cell bank as previously described [36].

### 4.4. Immunofluorescence

iPS-derived RPE cells were thawed and seeded onto Matrigel-coated Permanox-treated 8-chambered slide. On day 30, cells were washed with phosphate-buffered saline (PBS) and fixed in 4% paraformaldehyde (PFA) in 0.1 M sodium cacodylate buffer (pH 7.4) for 15 min at 4 °C. Cells were washed twice with cold PBS and then blocked with 5% bovine serum albumin (BSA) (Millipore Sigma) with 0.2% Triton X-100 (Millipore Sigma) to permeabilize the cell membrane. After blocking, cells were incubated overnight at 4 °C with primary antibody (Table S1). Cells were washed three times with cold PBS, incubated with the corresponding secondary antibody (Table S2) conjugated to AlexaFlour (1:300 dilution) (Invitrogen) or Cy2, 3, or 5 (1:200 dilution) (Jackson-Immuno) for 1 h at 4 °C, incubated with Hoechst (2 μg/mL)(Invitrogen) for 5 min at room temperature, washed three times with PBS, mounted with 80 μL Prolong Gold Mountant (Invitrogen) and coverslip, and imaged on Olympus IX70 Inverted Compound microscope (Tokyo, Japan).

### 4.5. Quantitative PCR Analysis

Passage 3 iPS-derived RPE cells were thawed and seeded at 1.5 × 10^5^ cells per cm^2^ onto Matrigel-coated 6-well plates (Corning). RPE cells were allowed to mature for 30 days and then were passaged using TrypLE (Gibco). Cells were collected by centrifugation at 2500× *g* for 5 min. Cells were lysed using Buffer RLT (RNEasy Mini Kit, Qiagen, Venlo, Netherlands) at a concentration of 350 μL per 1 × 10^6^ cells. cDNA was synthesized by two methods. Up to 1 μg of total RNA was used to synthesize cDNA using the iScript cDNA Synthesis Kit (Bio-Rad, Hercules, CA, USA, http://www.biorad.com/). Alternatively, 30 ng of RNA was used to synthesis cDNA using AgPath-ID™ One-Step RT-PCR Reagents. Primers used were TaqMan Gene Expression Assays (Thermo Fisher, Table S3). Quantitative real-time polymerase chain reaction was performed using CFX96™ Real-time PCR Detection System (Bio-Rad) using FAM detection. Twenty microliter reactions were run in triplicate in a 96-well plate. Data were normalized by two methods: the geometric mean of housekeeping genes *SERF2*, *EIF2B2*, and *UBE2R2* or by the Livak method [52].

### 4.6. ELISA for PEDF and MFG-E8

Passage 3 iPS-derived RPE, fetal RPE, ARPE-19, and RMECs were thawed and seeded at 1.5 × 10^5^ cells per cm^2^ in triplicate onto Matrigel-coated 24-well Transwell® inserts (Corning). Cells were allowed to mature for 30 days and the medium was collected 48 h after the last medium change from the apical to basal compartments. Media samples were then flash frozen in liquid nitrogen and stored at −80 °C. Secreted protein was measured using ELISA as per manufacturer’s recommendations for pigment-epithelial-derived factor (Human PEDF ELISA Kit, BioProductsMD, Middletown, MD, USA, http://www.bioproductsmd.com/) and milk-fat globule-EGF factor 8 (Human MFG-E8 Quantikine ELISA Kit, R&D Systems, Minneapolis, MN, USA, http://rndsystmes.com/). Optical density was measured using a fluorescent plate reader (Synergy H1 Hybrid Multi-Mode Reader).

### 4.7. Phagocytosis Assay

Passage 3 iPS-derived RPE, fetal RPE, ARPE-19, and RMECs were thawed and seeded at 1.5 × 10^5^ cells per cm^2^ in quadruplicate onto Matrigel-coated 96 well plates (clear bottom, black walls) (Corning). Cells were allowed to mature for 30 days and then were challenged with approximately 10 FITC-labeled (Invitrogen) photoreceptor outer segments (POS) (InVision BioResources, Seattle, WA, USA, http://www.invisionbio.com/) per cell for 5 h as previously described and utilized in disease modeling [35,53,54]. Excess POSs were aspirated and the cells were washed for 1 min three times with room-temperature PBS. Subsets of samples were treated with 0.4% Trypan blue (Fisher Scientific) for 10 min at room temperature to quench FITC fluorescence. All samples were washed twice with PBS, fixed with ice-cold 100% methanol (Millipore Sigma), and rehydrated with PBS for overnight incubation. Fluorescence was quantified using FITC detection (excitation: 488 nm, detection: 520 nm) (Synergy H1 Hybrid Multi-Mode Reader, BioTek, Winooski, VT, USA, http://biotek.com/), as previously described and utilized in disease modeling [35,40,55].

### 4.8. Western Blot Analysis

iPS-derived RPE cells were thawed and seeded at 1.5 × 10^5^ cells per cm^2^ in duplicate on Matrigel-coated 6-well plates (Corning). RPE cells were allowed to mature for 30 days and then were passaged using TrypLE (Gibco). An average of 1.5 × 10^6^ cells were collected from each well and pelleted by centrifugation at 2500× *g* for 5 min. The supernatant was discarded, and the cells were washed twice in cold PBS and pelleted by centrifugation. Cells were lysed using RIPA lysis buffer (Thermo Scientific) at a concentration of 5 × 10^6^ cells per mL as per manufacturer’s recommendations, with 1× final concentration of Halt Protease Inhibitor Cocktail and Halt Phosphatase Inhibitor Cocktail (Thermo Scientific). The cell buffer mixture was shaken gently on an orbital shaker for 15 min at 4 °C and then centrifuged at 14,000× *g* for 15 min. The supernatant was collected and stored at −80 °C.

The protein concentration was determined using a Pierce BCA Protein Assay Kit (Thermo Fisher Scientific) according to the manufacturer’s microplate procedure (Synergy H1 Hybrid Multi-Mode Reader, BioTek, Winooski, VT, USA, http://www.biotek.com/). Cell lysates (10–20 μg total protein per lane) were separated by sodium dodecyl sulfate polyacrylamide gel electrophoreses (SDS-PAGE) in NovexTM WedgeWellTM Tris-Glycine Gels (Invitrogen). Separated proteins were transferred to nitrocellulose membranes with a 0.45 μm pore size (Life Technologies, Gaithersburg, MD, USA) using PierceTM Power Blotter semi-dry transfer, blocked for 1 h at room temperature in blocking buffer for Fluorescent Western Blotting (Rockland, Pottstown, PA, USA, http://www.rockland-inc.com/), and incubated with primary antibodies overnight at 4 °C: monoclonal mouse anti-GAPDH loading control (0.5 μg/mL; MA5-15738); polyclonal rabbit anti-PRPF8 (1 μg/mL; ab87433 and ab79237). Membranes were washed with Tris-Buffered Saline Tween-20 (TBST) (Thermo Fisher Scientific), probed with secondary antibodies for 30 min at room temperature: donkey anti-mouse IgG (H + L) (IRDye^®^ 680RD; 0.06 ng/mL; LI-COR Biosciences, Lincoln, NE, USA, http://www.licor.com/) and donkey anti-rabbit IgG (H + L) (IRDye^®^ 800CW; 0.06 ng/mL), and washed with TBST. The fluorescent signal was visualized on an Odyssey Imager (LI-COR Biosciences).

### 4.9. Statistical Analysis

For all experiments, two-way ANOVA tests were performed using GraphPad Prism. *N* of 3 indicates 3 independently differentiated RPE cell lines for diseased, corrected, and wild-type cells. Significance was determined at *p* < 0.05.

## 5. Conclusions

Our findings demonstrate that mature, functional RPE cells can be differentiated reproducibly from six iPSC lines. This work shows that previous reports of phagocytic defects in mouse RPE are not detected in a human model of RP13. This is the first report of MFG-E8 secretion by patient-derived RPE cells. Measurements of PEDF secretion revealed that the diseased RPE cells may have generated a less robust barrier, which is in line with previous publications regarding pathology in RPE cells. Due to the fact that 5–46% of phenotypic variability is caused by natural genetic variation, the future of disease modelling relies on the establishment of robust protocols for producing homogeneous populations of cells. Next-generation sequencing may help elucidate genetic differences between the diseased and gene-edited cells. Future studies should focus on extensive investigation of the entire transcriptome to elucidate variations in alternative splicing in the context of RP13. While the molecular pathology of splicing factor retinitis pigmentosa is still under investigation, our results provide a baseline for establishing a homogeneous population of cells for subsequent disease modeling.

## Supplementary Materials

Supplementary materials can be found at http://www.mdpi.com/1422-0067/19/12/4127/s1.

## Abbreviations

ARPE-19	adult retinal pigment epithelial cells from a 19-year-old donor
BEST1/*BEST1*	bestrophin 1 (protein/gene)
CRISPR/Cas9	clustered regularly interspaced short palindromic repeats/CRISPR-associated system 9
fRPE	fetal retinal pigment epithelial cells
hESC	human embryonic stem cells
iPSC	induced pluripotent stem cells
MFG-E8	milk-fat globule-EGF factor 8
PEDF	pigment-epithelial-derived factor
PMEL/PMEL17	premelanosome 17 (protein/gene)
POS	photoreceptor outer segment
PRPF8	pre-mRNA processing factor 8
RMEC	retinal microvascular endothelial cells
RP13	retinitis pigmentosa 13
RPE	retinal pigment epithelial cells
SD	standard deviation
