# Supplementary material for: Functional Assessment of Patient-Derived Retinal Pigment Epithelial Cells Edited by CRISPR/Cas9
Source: Int J Mol Sci. 2018 Dec 19;19(12):4127. doi: 10.3390/ijms19124127 (PMC6321630; doi:10.3390/ijms19124127)
Supplement: Supplementary file 1 [file ijms-19-04127-s001.pdf]

Supplementary Materials

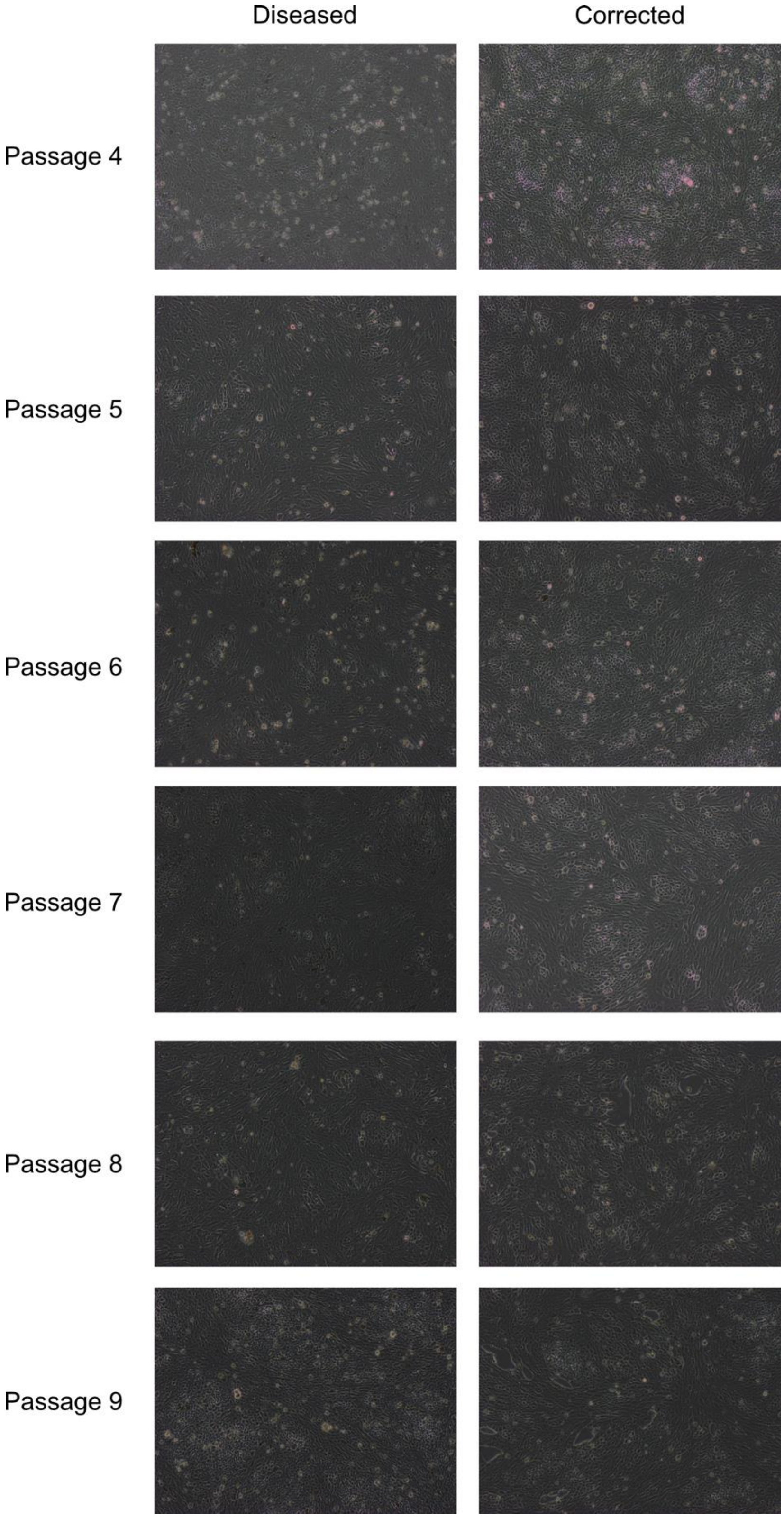

Figure S1. Atrophy of RPE upon extended passage, maintenance of polygonal morphology (10× objective).

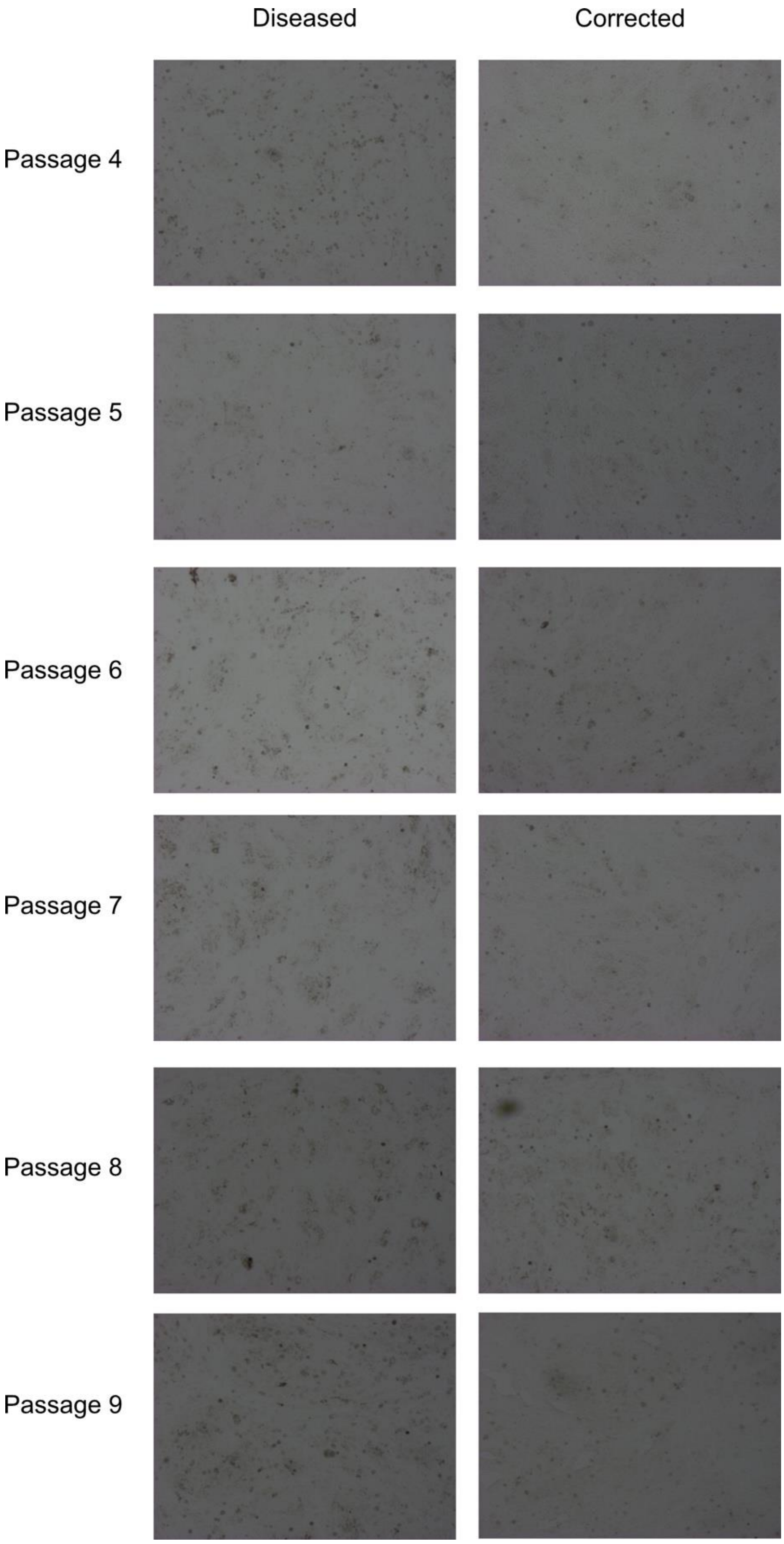

Figure S2. Atrophy of RPE upon extended passage, maintenance of pigmentation (10× objective).

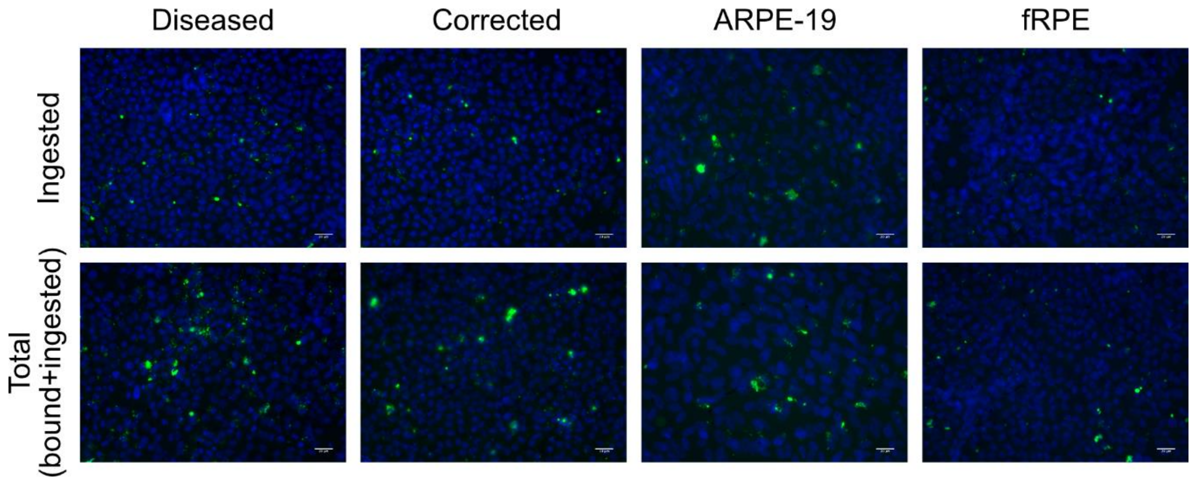

Figure S3. Representative images of bound and ingested FITC-labeled POS in RPE (10× objective). Scale bar equals 20 µm.

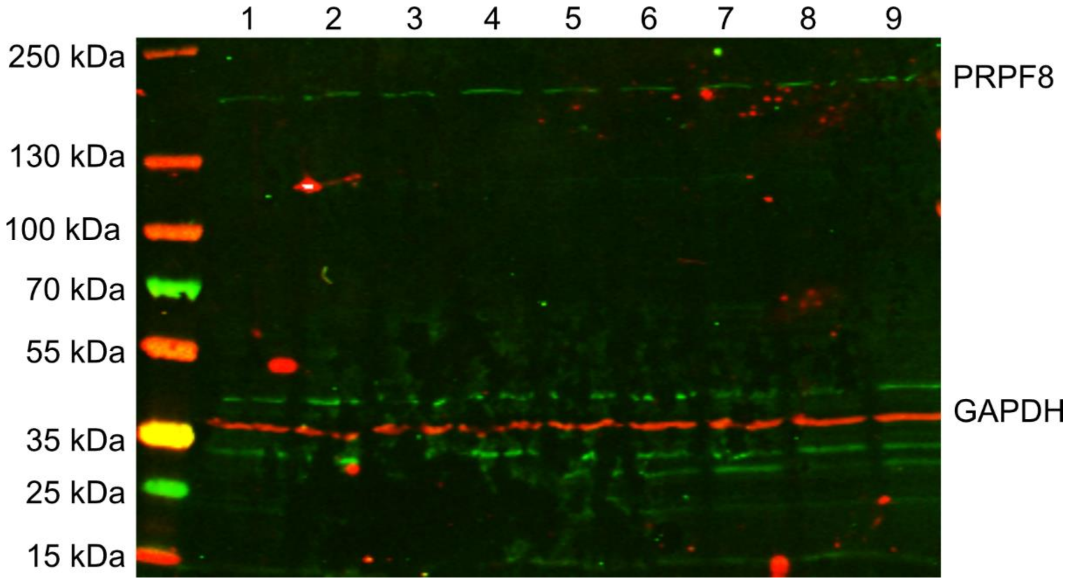

Figure S4. Western blot detection of PRPF8 and GAPDH loading control from passage 3 day 30 stem cell-derived RPE. (1–3) diseased clones, (4–6) corrected clones, (7–9) wild-type H9, UCSF4, and MyCell, respectively. 10 µg of protein per lane.

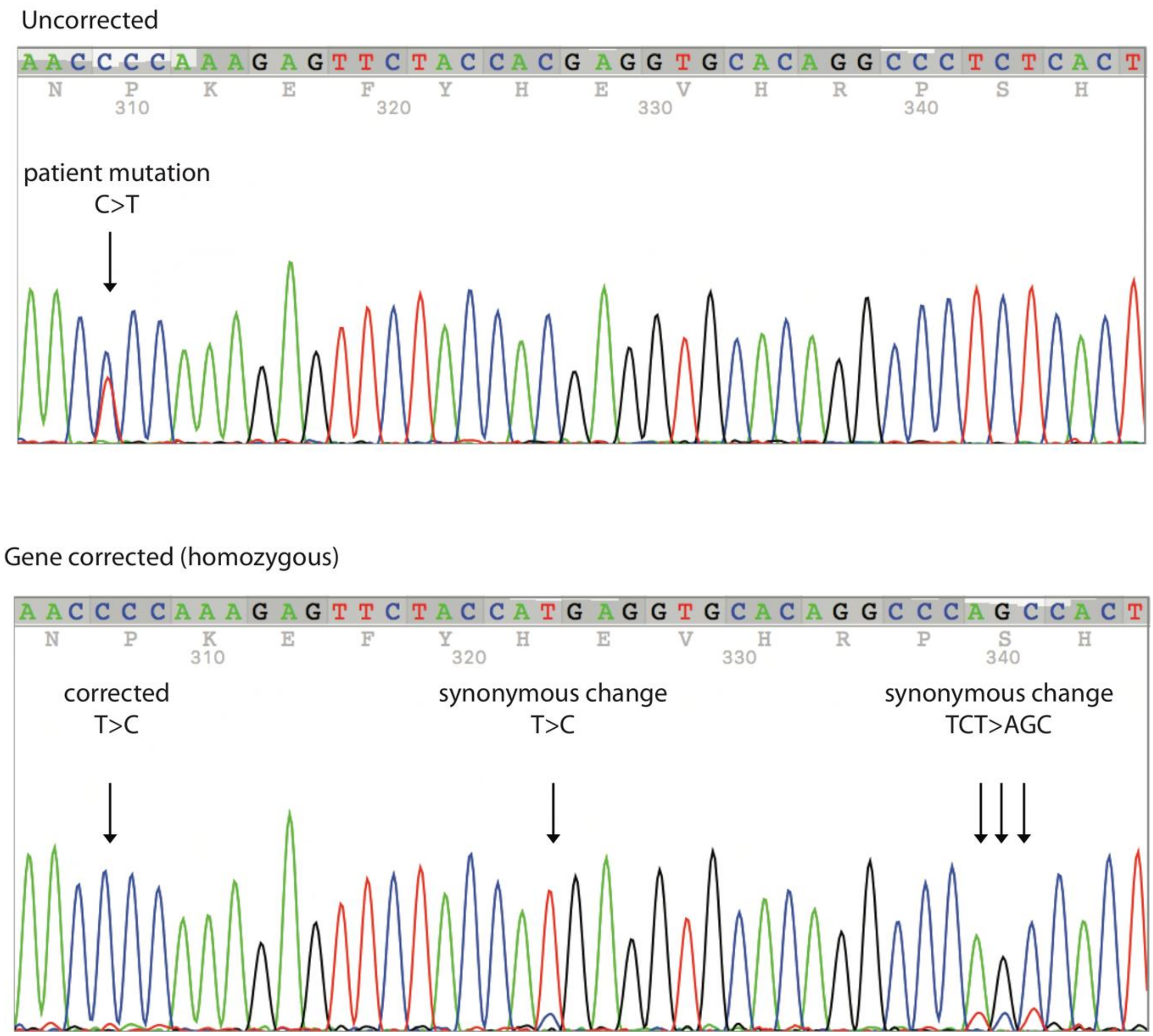

Figure S5. Confirmation of *PRPF8* gene-correction. mRNA encoding Cas9-Gem and a ssODN repair template was introduced into patient-specific iPSC via electroporation.

Table S1. Primary Antibodies.

Target Protein	Clonality/Immunoglobulin	Host	Dilution	Source/ Catalog Number
PMEL17	Monoclonal	Mouse	1:100	Dako/M0634
PRPF8	Polyclonal	Rabbit	1:100	abcam/ab87433, ab79237
ZO-1	Polyclonal	Rabbit	1:100	abcam/ab59720
BEST1	Monoclonal/Igg1	Mouse	1:100	abcam/ab2182
GAPDH	Monoclonal/Igg1	Mouse	1:100	ThermoFisher/MA5-15738

Table S2. Secondary Antibodies.

Target	Fluorophore/Immunoglobulin	Host	Dilution	Source/Catalog Number
Anti-rabbit	Cy2/IgG (H+L)	Donkey	1:300	Jackson Immuno/711-225-152
Anti-mouse	Cy3	Donkey	1:300	Jackson Immuno/715-165-150
Anti-rabbit	Cy3	Donkey	1:300	Jackson Immuno/711-165-752
Anti-mouse	800CW	Donkey	1:10,000	LI-COR/925-32212
Anti-rabbit	680RD	Donkey	1:10,000	LI-COR/925-68073

Table S3. Taqman Probes.

Target	Assay ID Number
RPE-specific protein 65 kDa (*RPE65*)	Hs01071462_m1
Bestrophin 1 (*BEST1*)	Hs00188249_m1
Retinaldehyde binding protein 1 (*RLBP1*)	Hs00165632_m1
Microphthalmia-associated transcription factor (*MITF*) isoform 2	AJD1S3G
Tyrosinase (*TYR*)	Hs00165976_m1
Paired box 6 (*PAX6*)	Hs01088112_m1
Pan microphthalmia-associated transcription factor (*MITF*)	Hs01117293_m1
Zinc finger protein 42 (*REX1*)	Hs01124465_m1
Splat-like transcription factor 4 (*SALL4*)	Hs00360675_m1
S100 calcium binding protein A4 (*S100A4*)	Hs00243202_m1
Integrin, α 2 (*ITGA2*)	Hs00158127_m1
Microphthalmia-associated transcription factor (*MITF*) isoform 4+5	Hs01117294_m1
Platelet/endothelial cell adhesion molecule 1 (*PECAM1*)	Hs00169777_m1
Microtubule-associated protein 2 (*MAP2*)	Hs00258900_m1
Marker of proliferation Ki-67 (*MKI67*)	Hs01032443_m1
Tyrosinase-related protein 1 (*TYRP1*)	Hs00167051_m1
Premelanosome (*PMEL*)	Hs00173854_m1
Eukaryotic translation initiation factor 2B, subunit 2 β (*EIF2B2*)	Hs00204540_m1
Ubiquitin-conjugating enzyme E2R 2 (*UBE2R2*)	Hs00215107_m1
Small EDRK-rich factor 2 (*SERF2*)	Hs00428481_m1
